# Lung Screen Uptake Trial: results from a single lung cancer screening round

**DOI:** 10.1136/thoraxjnl-2020-214703

**Published:** 2020-08-05

**Authors:** Mamta Ruparel, Samantha L Quaife, Jennifer L Dickson, Carolyn Horst, Sophie Tisi, Helen Hall, Magali Taylor, Asia Ahmed, Penny Shaw, Stephen Burke, May-Jan Soo, Arjun Nair, Anand Devaraj, Karen Sennett, Stephen W Duffy, Neal Navani, Angshu Bhowmik, David R Baldwin, Sam M Janes

**Affiliations:** 1 Lungs for Living Research Centre, UCL Respiratory, University College London, London, England, UK; 2 Research Department of Behavioural Science and Health, University College London, London, UK; 3 Department of Radiology, University College London Hospitals NHS Foundation Trust, London, London, UK; 4 Department of Radiology, Homerton University Hospital NHS Foundation Trust, London, London, UK; 5 Department of Radiology, Royal Brompton Hospital, London, UK; 6 Killick Street Health Centre, London, UK; 7 Wolfson Institute of Preventive Medicine, Barts and London, London, UK; 8 Thoracic Medicine Department, University College London Hospitals NHS Foundation Trust, London, London, UK; 9 Respiratory Medicine, Homerton University Hospital NHS Foundation Trust, London, London, UK; 10 Respiratory Medicine Unit, David Evans Research Centre, Nottingham University Hospitals NHS Trust, Nottingham, Nottinghamshire, UK

**Keywords:** lung cancer, imaging/CT MRI etc

## Abstract

The Lung Screen Uptake Trial tested a novel invitation strategy to improve uptake and reduce socioeconomic and smoking-related inequalities in lung cancer screening (LCS) participation. It provides one of the first UK-based ‘real-world’ LCS cohorts. Of 2012 invited, 1058 (52.6%) attended a ‘lung health check’. 768/996 (77.1%) in the present analysis underwent a low-dose CT scan. 92 (11.9%) and 33 (4.3%) participants had indeterminate pulmonary nodules requiring 3-month and 12-month surveillance, respectively; 36 lung cancers (4.7%) were diagnosed (median follow-up: 1044 days). 72.2% of lung cancers were stage I/II and 79.4% of non-small cell lung cancer had curative-intent treatment.

## Introduction

Lung cancer screening (LCS) by low-dose CT (LDCT) has been repeatedly shown in clinical trials to reduce lung cancer mortality.^1–3^ The benefits of screening may be underestimated in these trials due to participants being younger, of higher socioeconomic position and disproportionately former rather than current smokers compared with the high-risk target population. The risk profile of the population enrolled determines the prevalence and stage of lung cancers, the false positive rate and the mortality benefit. Screening the highest risk quintiles can optimise the benefit-harm ratio while making LCS more equitable, efficient and cost-effective.^4 5^

Data from a prior UK-based ‘real world’ screening pilot in Manchester has shown compelling results with high levels of attendance by those from lower socioeconomic quintiles and radical treatment rates.^6^ Here we report the nodule and cancer outcomes from the Lung Screen Uptake Trial (LSUT).

## Methods

The LSUT methods and primary attendance results have been described previously^7 8^ and more detail is included in the online supplementary appendix. LSUT was a randomised controlled trial evaluating the impact of ‘targeted, stepped and low burden’ invitation materials on attendance of a ‘lung health check’ (LHC) appointment. Individuals aged 60 to 75 years, who had been recorded as ‘current smokers’ within the seven preceding years were sent an invitation letter from their usual general practice doctor inviting them to an LHC. Those attending were invited to participate in the study, and those meeting any of the following criteria were offered a single LDCT on the same day (or later if preferred): ≥30 pack-years and if a former smoker had quit ≤15 years ago, or a lung cancer risk of ≥1.51% or ≥2.5% as determined by the Prostate, Lung, Colorectal and Ovarian study or the Liverpool Lung Project models, respectively.

10.1136/thoraxjnl-2020-214703.supp1 Supplementary data

Self-reported demographics, smoking, family and medical history were recorded prospectively. Hand-held pre-bronchodilator spirometry, height, weight and blood pressure were recorded. LDCT findings were evaluated and managed in accordance with the British Thoracic Society (BTS) 2015 guidelines for pulmonary nodules^9^ and the National Institute for Health and Care Excellence (NICE) guidelines for the diagnosis and management of lung cancer.^10^ Staging was carried out according to the 7^th^ edition TNM (tumour, node, metastases) classification system.

In the present study, we report the outcomes relating to LDCT scans with an indeterminate pulmonary nodule or suspected lung cancer. Other incidental finding outcomes have been reported elsewhere.^11 12^ Study participants with complete smoking and lung cancer risk data were included. Descriptive statistics were used to present the data pertaining to pulmonary nodules and lung cancer outcomes.

## Results

**Figure 1:**
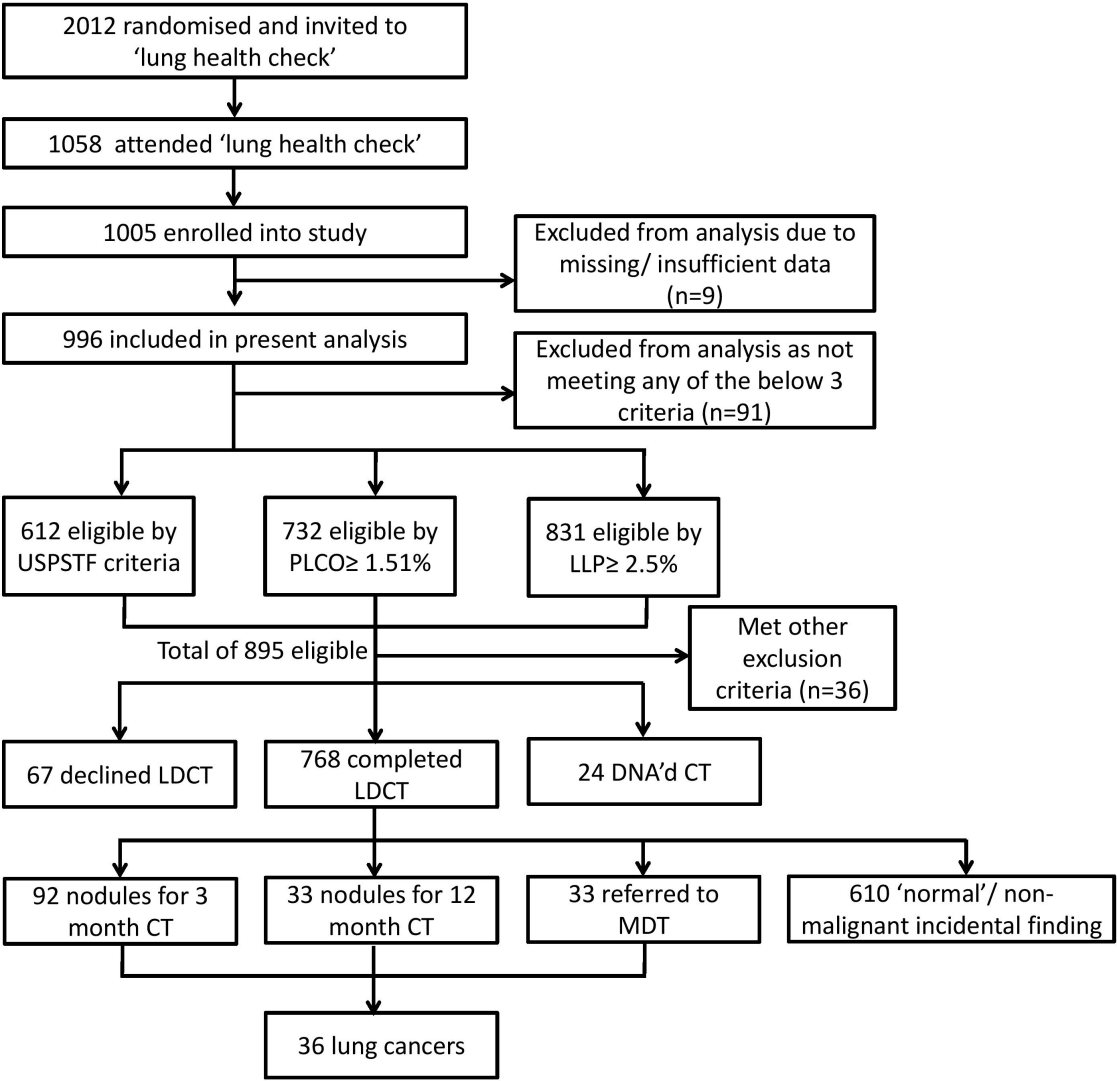
Flow chart of invitees and participants demonstrating numbers identified, invited, enrolled, eligible for LDCT and that completed a LDCT examination. DNA, did not attend; LDCT, low-dose CT; LLP, Liverpool Lung Project; MDT, multidisciplinary team; PLCO_m2012_, Prostate, Lung, Colorectal and Ovarianstudy model 2012; USPSFT, United States Preventive Services Task Force.

Of the 1058 (52.6%) invitees (n=2012) attending a LHC appointment between November 2015 and July 2017, 996 were included in the present analysis. A total of 895 participants were eligible for LDCT, though 36 were excluded due to prior CT of the chest in the past year, or an inability to lie flat and 91 participants declined or failed to attend the CT. An LDCT examination was completed by 768 (77.1%) of the participants (figure 1). The demographic characteristics of the 996 participants included are presented in table 1.

**Table 1:** Participant characteristics by group (% totals may not sum up due to rounding or missing data)

Variables	No LDCT n=228 median (IQR) or n (%)	No lung cancer n=732 median (IQR) or n (%)	Lung cancers n=36 median (IQR) or n (%)	All groups n=996 median (IQR) or n (%)
**Age (in years**)				
60–63	86 (37.7)	241 (32.9)	8 (22.2)	335 (33.6)
64–67	72 (31.6)	238 (32.5)	11 (30.6)	321 (32.2)
68–72	48 (21.1)	158 (21.6)	13 (36.1)	219 (22.0)
73–76	22 (9.7)	95 (13.0)	4 (11.1)	121 (12.2)
**Gender**				
Female	109 (47.8)	317 (43.3)	23 (63.9)	449 (45.1)
**Ethnicity**				
White	183 (80.3)	607 (82.9)	34 (94.4)	824 (82.7)
Black/African/Caribbean	23 (10.1)	77 (10.5)	1 (2.8)	101 (10.1)
Other	22 (9.7)	48 (6.6)	1 (2.8)	71 (7.1)
**Highest level of education**				
Left school at or before age 15	105 (46.1)	395 (54.0)	20 (55.6)	520 (52.2)
GCSEs, O-levels or equivalent	26 (11.4)	75 (10.3)	3 (8.3)	104 10.4)
A-levels or equivalent	24 (10.5)	70 (9.6)	4 (11.1)	98 (9.8)
Further education	14 (6.1)	31 (4.2)	3 (8.3)	48 (4.8)
Bachelor degree	34 (14.9)	84 (11.5)	2 (5.6)	120 (12.1)
Further higher degree	20 (8.8)	64 (8.7)	4 (11.1)	88 (8.8)
Other	5 (2.2)	13 (1.8)	0 (0)	18 (1.8)
**Index of Multiple Deprivation quintile**				
1 (most deprived)	117 (51.3)	402 (54.9)	19 (52.8)	538 (54.0)
2	87 (38.2)	245 (33.5)	12 (33.3)	344 (34.5)
3	3 (1.3)	17 (2.3)	1 (2.8)	21 (2.1)
4	0 (0)	2 (0.3)	0 (0)	2 (0.2)
5 (least deprived)	0 (0)	0 (0)	0 (0)	0 (0)
**Smoking history**				
Current smoker	148 (64.9)	527 (72.0)	31 (86.1)	706 (70.9)
Years smoked (years)	42 (33 to 51)	47 (44 to 51)	51 (47 to 54)	47 (42 to 51)
Years quit (years)	0 (0 to 3)	0 (0 to 0)	0 (0 to 0)	0 (0 to 0)
Average smoking intensity (cigs/day)	14 (8 to 20)	20 (10 to 20)	20 (10 to 23)	17 (10 to 20)
Pack years	23 (10 to 41)	38 (26 to 51)	46 (26 to 63)	36 (21 to 50)
**Lung cancer risk**				
PLCO (% 6-year risk)	1.40 (0.39 to 5.48)	3.74 (1.80 to 7.14)	5.68 (2.96 to 9.27)	3.43 (1.38 to 6.97)
LLP (% 5-year risk)	3.07 (1.55 to 7.16)	5.58 (3.79 to 8.75)	5.5 (4.58 to 9.77)	5.20 (3.16 to 8.56)
**Physical measurements**				
FEV1 (l/min)	2.12 (1.68 to 2.57)	2.06 (1.64 to 2.56)	1.74 (1.12 to 2.2)	2.06 (1.64 to 2.55)
FEV1 (% predicted)	85 (69 to 98)	82 (66 to 96)	73 (53 to 89)	82 (67 to 97)
FEV/FVC (%)	70 (63 to 77)	69 (61 to 75)	62 (54 to 69)	69 (62 to 76)
BMI (kg/m^2^)	25.8 (22.9 to 29.1)	26.2 (23 to 29.4)	23.5 (22.5 to 26)	26.0 (22.9 to 29.2)
**WHO Performance Status**				
0 - asymptomatic	203 (89.0)	660 (90.2)	28 (77.8)	891 (89.5)
1 - completely ambulatory	23 (10.1)	64 (8.7)	8 (22.2)	95 (9.5)
2 - <50% of day in chair/ bed	1 (0.4)	8 (1.1)	0 (0)	9 (0.9)
3 - >50% of day in chair/ bed	1 (0.4)	0 (0)	0 (0)	1 (0.1)
**LDCT**				
Follow-up duration since LDCT (days)	n/a	1007 (851 to 1143)	1044 (933 to 1153)	1008 (853 to 1144)

BMI, body mass index; cigs, cigarettes; CT, Computed Tomography scan; GCSE, General Certificate of Secondary Education; LDCT, low-dose CT; LLP, Liverpool Lung Project; PLCO, Prostate, Lung, Colorectal and Ovarian study; USPSTF, United States Preventive Services Task Force; VATS, Video Assisted Thoracoscopic Surgery.

At the baseline LDCT scan, a total of 125/768 participants had indeterminate pulmonary nodules requiring 3-month (n=92 (11.9%)) or 12-month (n=33 (4.3%)) surveillance and a further 33 (4.3%) were considered to have lesions suspicious for lung cancer that instigated referral to the local multidisciplinary meeting. The remaining 610 participants had a ‘normal’ scan or had non-malignant findings that have been discussed elsewhere.^13 14^ After a median follow-up of 1044 days, a total of 36 lung cancers (4.7%) were diagnosed. Of these, 17 (*51.5% of those referred to the lung cancer clinic*) were diagnosed directly following the baseline LDCT and the remainder were diagnosed following further surveillance CT scans of indeterminate nodules in the 3-month (n=16, 17.4% of nodules in this group) or 12-month surveillance groups (n=3, 9.1% of nodules in this group).

For invasive investigations we report the data as a percentage of the total number of lung cancers (table 2). Forty-nine (136%) participants underwent positron emission tomography scan, 10 (27.8%) had endobronchial ultrasound and 5 (13.9%) underwent percutaneous CT-guided lung biopsy. Numbers of diagnostic investigations performed in those without a later diagnosis of cancer are also detailed in table 2. Of note, there were no adverse outcomes from diagnostic investigations in this group. Twenty-one (58.3%) participants had a surgical resection without prior histological confirmation of malignancy (and underwent frozen section at the time of the resection), though some had undergone diagnostic staging examinations prior to surgery. 2 out of 28 (7.1%) lung resections were subsequently found to be benign and this represented 0.3% of participants without lung cancer. There were no deaths within 90 days of surgery.

**Table 2:** Investigations rates, and stage, histology and treatments from the baseline LDCT scan

	Number in total cohort (% of total lung cancers, n=36 (*except treatments)	Number among those without a diagnosis of lung cancer (% of total participants without lung cancer, n=732)
**Diagnostic or staging investigations**		
Positron emission tomography (PET)	49 (136)	16 (2.2)
Percutaneous non-lung biopsy	5 (13.9)	0 (0)
Other percutaneous biopsy	6 (16.7)	1 (0.1)
Cervical lymph node FNA	2 (5.6)	0 (0)
Fibreoptic bronchoscopy	12 (33.3)	9 (1.2)
Endobronchial ultrasound	10 (27.8)	1 (0.1)
Endoscopic ultrasound	1 (2.8)	0 (0)
VATS or open lung biopsy	21 (58.3)	2 (0.3)
Total: PET or invasive procedures		29 (4.0)
**Histology**		
Invasive adenocarcinoma	16 (44.4)	
Minimally invasive adenocarcinoma	3 (8.3)	
Adenocarcinoma in situ	1 (2.8)	
Squamous cell carcinoma	6 (16.7)	
Mixed NSCLC (ie, adenosquamous)	2 (5.6)	
Small cell lung cancer	2 (5.6)	
Multiple or mixed histology (small cell + NSCLC)	3 (8.3)	
Radiological diagnosis	2 (5.6)	
Carcinoid	1 (2.8)	
**Stage (TNM 7^th^ edition**)		
Stage I & II	26 (72.2)	
Ia	22 (61.1)	
Ib	1 (2.8)	
IIa	3 (8.3)	
IIb	0 (0)	
IIIa	6 (16.7)	
IIIb	1 (2.8)	
IV	3 (8.3)	
Treatments (NSCLC) (*% are of total NSCLC)		
Curative intent	27 (79.4)	
Sub-lobar resection	11 (32.4)	
Lobectomy	15 (44.1)	
SABR	1 (2.9)	
Concurrent chemoradiation	2 (5.9)	
Palliative chemotherapy±radiation	4 (11.8)	
Surveillance	1 (2.9)	
Treatments (SCLC) (*% are of total SCLC)		
Radical chemoradiation	2 (100)	

CT, CT scan; DNA, did not attend; FNA, fine needle aspiration; GCSE, General Certificate of Secondary Education; LDCT, low-dose CT; LHC, lung health check; MDT, multidisciplinary team; NSCLC, non-small cell lung cancer; SABR, stereotactic ablative radiotherapy; SCLC, small cell lung cancer; TNM, tumour, node, metastases; UKLS, United Kingdom Lung Cancer Screening Trial; USPSTF, United States Preventive Services Task Force; VATS, video assisted thorascopic surgery.

Twenty-six (72.2%) of all lung cancers were stage I or II and 27 (79.4%) of those with non-small cell lung cancer (NSCLC) had curative-intent treatment (including sublobar resection, lobectomy and stereotactic ablative radiotherapy). Of the two participants with small cell lung cancer, both received concurrent chemoradiation. Ten (27.8%) participants had advanced stage (III or IV) disease, resulting in four (11.8%) of those with NSCLC undergoing palliative chemotherapy or radiotherapy (table 2). Online supplementary table e1 presents details on all 36 lung cancers.

## Discussion

This observational cohort study demonstrated that despite the very high risk of lung cancer in the cohort, 75.0% of lung cancers detected were early stage and 79.4% of the patients with NSCLC had treatment with curative intent. Indeterminate pulmonary nodules for 3-month and 12-month surveillance were detected in 11.9% and 4.3% of the participants screened, respectively, and lung cancer was detected in 4.7%.

The rate of indeterminate pulmonary nodules (16.2%) was lower than in NLST (National Lung Screening Trial; 24.2%)^1^ and NELSON trial (19.2%).^15^ This may have been in part due to implementation of the 2015 BTS pulmonary nodule guidelines which enables a more conservative approach to nodules smaller than 5 mm.^9^ The lung cancer prevalence was significantly higher than the majority of LCS trials, which have reported a 1% to 2% prevalence.^1 16 17^ However other higher-risk LCS cohorts have demonstrated a similar lung cancer prevalence to that seen here.^6 18^ The proportion of participants with early-stage lung cancer who received treatment with curative intent was slightly lower than observed in UKLS,^17^ which again may reflect the population screened. The number of invasive tests for those without a diagnosis of lung cancer was low, with only 4% of individuals without cancer having a positron emissiontomography-CT (PET-CT) scan or other invasive tests such as bronchoscopy or percutaneous biopsy.

A strength of this study is that it demonstrates a method of recruiting otherwise underserved populations as evidenced by the low socioeconomic and education levels in the majority of the cohort and as such this study illustrates a pragmatic, ‘real-world’ approach to LCS. It is limited by the small sample size and low number of cancers. We acknowledge that this cohort had particularly high lung cancer risk, however, in light of emerging evidence advocating risk-based selection of LCS-eligible individuals,^4 19^ we believe the findings reported here are generalisable to the LCS-eligible population.

In conclusion, the rate of indeterminate pulmonary nodules was lower and the rate of lung cancer was higher than previous randomised LCS trials, and one in six individuals with an indeterminate nodule requiring 3-month surveillance LDCT were subsequently diagnosed with lung cancer. From these findings, as well as the impressive early-stage disease and curative intent treatment rates observed, we propose that LCS in a ‘real-world’ setting may be less harmful, more efficient and more cost-effective than has been seen in larger LCS studies.
