# Supplementary material for: Lung Screen Uptake Trial: results from a single lung cancer screening round
Source: Thorax. 2020 Aug 5;75(10):908–12. doi: 10.1136/thoraxjnl-2020-214703 (PMC7509385; doi:10.1136/thoraxjnl-2020-214703)
Supplement: Supplementary data [file thoraxjnl-2020-214703supp001.pdf]

Supplementary Appendix:

a) **Detailed Methods for the Lung Screen Uptake Trial**

b) **Table e1**

Detailed Methods for the Lung Screen Uptake Trial LDCT screening results

Participants, setting and study design

The LSUT methods have been described previously [1]. LSUT was a randomised study evaluating the impact of 'targeted, stepped, and low burden' invitation materials on attendance to a 'lung health check' (LHC) appointment [2]. Primary care records were searched to identify individuals aged between 60 and 75, who had been recorded as current smokers within the prior 7 years. Where multiple individuals cohabited at the same residence, only one was selected at random so as not to confound the primary research outcome. Individuals were also excluded if they had an active lung cancer diagnosis, cancer metastasis, were on palliative care treatment, had a lack of capacity to consent, or at the GP's discretion upon screening the final list of invitees. Either the control or intervention letter (randomly allocated) was sent on behalf of the individuals' primary care physician inviting individuals for an LHC at one of two London hospitals between November 2015 and July 2017. Individuals attending the LHC were invited to participate in the study.

Eligibility for LDCT screening

Those attending a Lung Health Check appointment (n=1057) who met the US Preventative Services Task Force (USPSTF) criteria for LCS (i.e. ≥30 pack-years and quit ≤15 years ago) [3], or a lung cancer risk of 1.51% as determined by the Prostate Lung Colorectal Ovarian study ($PLCO_{m2012}$) model [4] or 2.5% as determined by the Liverpool Lung Project (LLP) model [5], were offered a LDCT scan on the same day to screen for lung cancer (or could choose to have this later).

Data collection

Data were prospectively collected by a study practitioner at the LHC. Self-reported demographics (age, sex, ethnicity, education level, Index of Multiple Deprivation (IMD) score and rank), smoking, family and medical history were recorded. Hand-held spirometry, height, weight and blood pressure were recorded. Radiological data was recorded by the radiologist at the time of reporting the LDCT and clinical and pathological outcomes were recorded by a thoracic clinical research fellow. Data reported here are based on outcomes recorded up to two years after the last participant was recruited.

Outcome measures

PLCO and LLP scores

The publicly available algorithms for both of these risk prediction tools [5,6] were programmed into the study electronic database. The PLCO model was adapted for UK use. The ethnicity categories 'white', 'black' and 'other', used the coefficients from the PLCO model for 'white', 'black' and 'Asian' respectively. The education categories were also translated to match the UK education system with the same number of categories matching to a similar age or qualification.

LDCT

Participants undertook the examination via a 16-channel or higher multi-detector, non-ECG-voltage-gated CT without the administration of intravenous contrast. Imaging was performed during suspended maximal inspiration. The lung parenchyma (lung apices to bases) was scanned in its entirety in a single craniocaudal acquisition. The field of view (FOV) selected as the smallest diameter as measured from widest point of outer rib to outer rib large enough to accommodate the entire lung parenchyma. Thin detector collimation (0.5 mm) was used. Images were reconstructed at 0.5 -1.0 mm section thickness using standard soft tissue and lung algorithms. Radiation exposures were as low as possible whilst maintaining good image quality (median 1.2 mSv, IQR 0.9 mSv, 1.7 mSv). The tube potential and tube current-time product varied according to participant body habitus and were between 80-120 kVp and 20-80 mAs respectively.

Scans were single-read by a team of radiologists with expertise in thoracic CT reporting, and experience ranging from 5 to 28 years. Radiologists recorded details on up to two nodules, the total number of nodules seen, and any incidental findings. LDCT results were categorised into five categories. In the present study, we report the outcomes relating to LDCT scans with a radiologist-designated category of 'indeterminate pulmonary nodule' or 'suspicious of lung cancer'. Other outcomes from LDCT included scans with pulmonary or non-pulmonary incidental findings that may have required further assessment or treatment, or no significant findings, which were categorised separately and have been reported elsewhere [7,8]. For the purpose of this analysis, we have grouped these three into a single category of 'negative' scans.

Indeterminate pulmonary nodules

Indeterminate pulmonary nodules included those typically under 8mm or 300mm^3 that required a repeat scan at three or twelve months, as per the British Thoracic Society (BTS) 2015 guidelines for pulmonary nodules [9]. The BTS guidelines stipulate that nodules between 5 and 6mm diameter should have an annual surveillance CT and nodules between 6 and 8mm or 80 and 300mm^3 should have a CT at 3 and 12 months. Solid nodules confirmed to be stable on volumetry at 12 months or by diameter at 24 months may be discharged from surveillance. Sub-solid nodules require surveillance for 4 years. 'Positive' findings were referred to the local thoracic oncology multi-disciplinary team (MDT) for further assessment and were

managed according to the BTS guidelines for pulmonary nodules [9] and the National Institute for Health and Care Excellence (NICE) guidelines for the diagnosis and management of lung cancer [10].

Lung cancer

All subtypes of invasive and non-invasive lung cancer were included within the definition of lung cancer. These included neuroendocrine tumours including small cell and carcinoid; adenocarcinoma, including minimally invasive adenocarcinoma (MIA), and adenocarcinoma in situ (AIS); and other non-small cell lung cancers (NSCLC), including squamous cell, adeno-squamous and large cell carcinomas. Staging was carried out according to the 7th edition TNM classification system as this was the edition in use in participating thoracic oncology MDT meetings for the majority of the study.

Sample size & statistical analysis

The sample size for LSUT was based on the primary behavioural research question and is described in detail in the study's statistical analysis plan which were published prior to the commencement of the study [11]. The present analysis was a planned secondary analysis of the data. All study participants with complete smoking and lung cancer risk data were included. Participants were divided into three groups: those who did not have an LDCT, those who had an LDCT without lung cancer and those who had an LDCT who had a lung cancer diagnosed at some point during the follow up period. The follow up duration for participants varied by date of enrolment into the study, but was a minimum of 24 months from the last participant recruited. Descriptive statistics were used to present the data required to address the research questions above.

References:

1 Quaife SL, Ruparel M, Beeken RJ, *et al.* The Lung Screen Uptake Trial (LSUT): protocol for a randomised controlled demonstration lung cancer screening pilot testing a targeted invitation strategy for high risk and 'hard-to-reach' patients. *BMC Cancer* 2016;**16**:281. doi:10.1186/s12885-016-2316-z

2 Quaife SL, Ruparel M, Dickson JL, *et al.* Lung Screen Uptake Trial (LSUT): Randomised Controlled Trial Testing Targeted Invitation Materials. *Am J Respir Crit Care Med* 2019;:rccm.201905-0946OC. doi:10.1164/rccm.201905-0946OC

3 U.S. Preventive Services Task Force. U.S. Preventive Services Task Force: Final Recommendation Statement. 2013. http://www.uspreventiveservicestaskforce.org/uspstf13/lungcan/lungcanfinalrs.htm#summary (accessed 28 Nov 2014).

4 Tammemägi MC, Katki HA, Hocking WG, *et al.* Selection criteria for lung-cancer screening. *N Engl J Med* 2013;**368**:728–36. doi:10.1056/NEJMoa1211776

5 Cassidy A, Myles JP, van Tongeren M, *et al.* The LLP risk model: an individual risk prediction model for lung cancer. *Br J Cancer* 2008;**98**:270–6. doi:10.1038/sj.bjc.6604158

6 Brock University Lung Cancer Risk Calculators. https://brocku.ca/lung-cancer-screening-and-risk-prediction/risk-calculators/

7 Ruparel M, Quaife SL, Dickson JL, *et al.* Evaluation of cardiovascular risk in a lung cancer screening cohort. *Thorax* 2019;:thoraxjnl-2018-212812. doi:10.1136/thoraxjnl-2018-212812

8 Ruparel M, Quaife SL, Dickson JL, *et al.* Prevalence, Symptom Burden and Under-Diagnosis of COPD in a Lung Cancer Screening Cohort. 2019. www.atsjournals.org (accessed 7 Jun 2019).

9 Callister MEJE, Baldwin DR, Akram AR, *et al.* The British Thoracic Society guidelines on the investigation and management of pulmonary nodules. *Thorax* 2015;**70**:794–8. doi:10.1136/thoraxjnl-2015-207221

10 Baldwin DR, White B, Schmidt-Hansen M, *et al.* Diagnosis and treatment of lung cancer: summary of updated NICE guidance. *Br Med J* 2011;**342**:d2110. doi:10.1136/bmj.d2110

11 Quaife SL, Ruparel M, Duffy SW, *et al.* Lung Screen Uptake Trial (LSUT): Statistical Analysis Plan. OSF 2016. doi:10.17605/OSF.IO/9KUZB

Table e1. All 36 cancers as recorded at the beginning of July 2019

	Route to diagnosis	Diagnosis	Primary Treatment	Clinical TNM stage (pre-treatment)	Clinical stage	Pathological TNM stage	Pathological stage
1	Nodule follow up	Invasive adenocarcinoma	lobectomy	T1bN0M0	Stage 1A	pT1bN0M0 PL0 R0	Stage 1A
2	Nodule follow up	Squamous cell carcinoma	anatomical segmentectomy	T1aN0M0	Stage 1A	pT1aN0M0 PL0 R0	Stage 1A
3	Baseline LDCT	Squamous cell carcinoma	anatomical segmentectomy	T1aN0M0	Stage 1A	pT1aN0M0 PL0 R0	Stage 1A
4	Nodule follow up	Invasive adenocarcinoma	lobectomy	T1aN0M0	Stage 1A	pT1aN0M0 PL0 R0	Stage 1A
5	Baseline LDCT	Mixed NSCLC, i.e. adeno-squamous carcinoma	lobectomy	T1aN0M0	Stage 1A	pT1aN0M0 PL0 R0	Stage 1A
6	Baseline LDCT	Radiological diagnosis of lung cancer	SABR	T1bN0M0	Stage 1A	n/a	n/a
7	Nodule follow up	Minimally invasive adenocarcinoma	wedge resection	T1aN0M0	Stage 1A	pT1aN0M0 PL0 R0	Stage 1A
8	Nodule follow up	Invasive adenocarcinoma	anatomical segmentectomy	T1bN0M0	Stage 1A	pT1bN0M0 PL0 R0	Stage 1A
9	Nodule follow up	Invasive adenocarcinoma	anatomical segmentectomy	T1bN0M0	Stage 1A	pT1bN0M0 PL0 R0	Stage 1A
10	Nodule follow up	Squamous cell carcinoma	lobectomy	T1bN0M0	Stage 1A	pT1bN0M0 PL0 R0	Stage 1A
11	Nodule follow up	Invasive adenocarcinoma	wedge resection	T1aN0M0	Stage 1A	pT1aN0M0 PL0 R1	Stage 1A
12	Nodule follow up	Invasive adenocarcinoma	lobectomy	T1aN0M0	Stage 1A	pT1aN0M0 PL0 R1	Stage 1A
13	Baseline LDCT	Mixed or multiple histologies	anatomical segmentectomy	T1aN0M0	Stage 1A	pT1aN0M0 PL0 R0	Stage 1A
14	Nodule follow up	Invasive adenocarcinoma	anatomical segmentectomy	T1bN0M0	Stage 1A	pT1bN0M0 PL0 R0	Stage 1A

Table e1. (continued) All 36 cancers as recorded at the beginning of July 2019

	Route to diagnosis	Diagnosis	Primary Treatment	Clinical TNM stage (pre-treatment)	Clinical stage	Pathological TNM stage	Pathological stage
15	Nodule follow up	Mixed NSCLC, i.e. adeno-squamous carcinoma	lobectomy	T1aN0M0	Stage 1A	pT1aN0M0 PL0 R0	Stage 1A
16	Nodule follow up	Adenocarcinoma in situ	wedge resection	T1aN0M0	Stage 1A	pT1aN0M0 PL0 R0	Stage 1A
17	Nodule follow up	Minimally invasive adenocarcinoma	lobectomy	T1aN0M0	Stage 1A	pT1aN0M0 PL0 R0	Stage 1A
18	Nodule follow up	Minimally invasive adenocarcinoma	lobectomy	T1aN0M0	Stage 1A	pT1aN0M0 PL0 R0	Stage 1A
19	Nodule follow up	Squamous cell carcinoma	wedge resection	T1aN0M0	Stage 1A	pT1aN0M0 PL0 R0	Stage 1A
20	Nodule follow up	Invasive adenocarcinoma	lobectomy	T1aN0M0	Stage 1A	pT1aN0M0 PL0 R0	Stage 1A
21	Baseline LDCT	Carcinoid	wedge resection	T1aN0M0	Stage 1A	pT1aN0M0 PL0 R1	Stage 1A
22	Nodule follow up	Mixed or multiple histologies	lobectomy	T3N0M0	Stage 2B	pT3N0M0 PL1 R0	Stage 1A
23	Baseline LDCT	Radiological diagnosis of lung cancer	no treatment	T2aN0M0	Stage 1B	n/a	n/a
24	Baseline LDCT	Invasive adenocarcinoma	lobectomy	T2aN1M0	Stage 2A	pT2aN1M0 PL0 R0	Stage 2A
25	Baseline LDCT	Small cell carcinoma	concurrent chemoradiation	T1aN1M0	Stage 2A	n/a	n/a
26	Baseline LDCT	Squamous cell carcinoma	lobectomy	T1bN1M0	Stage 2A	pT1bN1M0 PL0 R0	Stage 2A
27	Baseline LDCT	Invasive adenocarcinoma	lobectomy	T2bN0M0	Stage 2A	pT4N1M0 PL2 R1	Stage 3A

Table e1. (continued) All 36 cancers as recorded at the beginning of July 2019

	Route to diagnosis	Diagnosis	Primary Treatment	Clinical TNM stage (pre-treatment)	Clinical stage	Pathological TNM stage	Pathological stage
28	Baseline LDCT	Invasive adenocarcinoma	lobectomy	T2aN2M0	Stage 3A	pT2aN2M0 PL0 R0	Stage 3A
29	Baseline LDCT	Squamous cell carcinoma	palliative radiotherapy	T2bN2M0	Stage 3A	n/a	n/a
30	Nodule follow up	Invasive adenocarcinoma	concurrent chemoradiation	T2aNM0	Stage 3A	n/a	n/a
31	Baseline LDCT	Invasive adenocarcinoma	concurrent chemoradiation	T1aN2M0	Stage 3A	n/a	n/a
32	Nodule follow up	Mixed or multiple histologies	lobectomy	T3N1M0	Stage 3A	pT3N1M0 PL0 R0	Stage 3A
33	Baseline LDCT	Small cell carcinoma	concurrent chemoradiation	T4N2M0	Stage 3B	n/a	n/a
34	Baseline LDCT	Invasive adenocarcinoma	palliative chemotherapy	T4N3M1b	Stage 4	n/a	n/a
35	Baseline LDCT	Invasive adenocarcinoma	palliative chemotherapy	T1aN2M1b	Stage 4	n/a	n/a
36	Baseline LDCT	Invasive adenocarcinoma	palliative chemotherapy	T4N3M1b	Stage 4	n/a	n/a
